# Exceptional response to TROP2 inhibition with sacituzumab govitecan in a patient with small cell carcinoma of the breast: a case report

**DOI:** 10.1177/17588359251322003

**Published:** 2025-02-26

**Authors:** Kasey C. Fitzsimmons, Michelle A. Fajardo, Zorawar Noor

**Affiliations:** David Geffen School of Medicine, University of California Los Angeles, CA, USA; Department of Pathology, Saddleback Memorial Medical Center, Laguna Hills, CA, USA; David Geffen School of Medicine, University of California Los Angeles, Los Angeles, CA, USA

**Keywords:** neuroendocrine tumor breast, sacituzumab govitecan-hziy, small cell carcinoma of the breast, TROP2 inhibition

## Abstract

We present a case report of a patient with metastatic small cell neuroendocrine carcinoma of the breast (SCNCB), a high-grade neuroendocrine triple-negative breast cancer, who achieved a complete response after two cycles of sacituzumab govitecan-hziy (SG) (Trodelvy) as third-line systemic therapy. She originally presented with estrogen receptor-positive disease, and we were able to clearly demonstrate transformation into triple-negative SCNCB via serial tissue biopsies. This is the first reported case of SG for SCNCB, and it shows an outstanding response in a patient who had undergone prior systemic therapies. Clinical trials are needed to address the potential role of TROP2 inhibition and the use of SG as a therapy for SCNCB.

## Plain language summary

Exceptional response with TROP2 inhibition in a patient with small cell carcinoma of the breast

A particularly rare subtype of breast cancer is primary small cell neuroendocrine carcinoma of the breast (SCNCB). Compared to other breast cancer subtypes, the small cell subtype is more aggressive and is associated with poorer outcomes. Current standard treatment of SCNCB mimics treatment used for small cell carcinoma of other organs, such as small cell lung cancer, which includes platinum chemotherapy combined with etoposide and immune checkpoint inhibition. However, treatment outcomes continue to remain poor, and there is a need to address further treatment options for SCNCB. Thus, we present the first reported case of sacituzumab govitecan-hziy (SG) (trodelvy) for SCNCB, showing outstanding response in a patient who had undergone prior systemic therapies. Further research is needed to address the potential role for TROP2 inhibition and the use of SG as a therapy for SCNCB.

## Introduction

A particularly rare subtype of breast cancer (BC) is primary small cell neuroendocrine carcinoma of the breast (SCNCB). Compared to other BC subtypes, the small cell subtype is more aggressive and is associated with poorer outcomes. The current standard treatment of SCNCB mimics treatment used for small cell carcinoma of other organs, such as small cell lung cancer (SCLC), which includes platinum chemotherapy combined with etoposide and immune checkpoint inhibition. However, treatment outcomes continue to remain poor, and there is a need to address further treatment options for SCNCB. We present a case of a patient with triple-negative breast cancer (TNBC) that transformed into SCNCB. The patient progressed on chemotherapy and immunotherapy as standard-of-care first-line therapy for metastatic TNBC, so she was started on sacituzumab govitecan-hziy (SG; Trodelvy, Gilead Sciences Inc., Foster City, USA) and had an outstanding response. Further research is needed to address the potential role of TROP2 inhibition and the use of SG as a therapy for SCNB.

## Case

A 54-year-old female presented with a gradually enlarging right breast mass with skin indentation, for approximately 3 years duration. She was diagnosed with clinical stage IIIA (cT2N2M0) invasive ductal carcinoma (IDC). It was Nottingham combined histologic grade 3 (9/9), estrogen receptor-positive (ER+; 80%), progesterone receptor positive (PR+; 70%), human epidermal growth factor receptor 2 negative (HER2 Neg; 0 by IHC) and had a Ki-67 proliferate rate of 48%. Baseline positron emission tomography (PET) was negative for distant metastatic disease. The patient preferred to avoid chemotherapy if possible, and she enrolled in a randomized controlled trial (TRIO B-038) in which all patients received the preoperative CDK4/6 inhibitor palbociclib (Ibrance, Pfizer, NY, USA) either in combination with the aromatase inhibitor, anastrozole, or in combination with an oral selective estrogen receptor degrader, giredestrant.^1^ She was randomized to the neoadjuvant palbociclib plus anastrozole arm, and she achieved a good partial response. However, 2 weeks prior to surgery, she noted rapid swelling in the right arm, new right breast induration, and worsening lymphedema in her arm; concerning for rapid progression. She underwent bilateral mastectomies with right axillary lymph node dissection, but her cancer was found to be partially unresectable. Pathology revealed treatment response in the breast, but no apparent treatment response in the axillary disease. Therefore, both areas were pathologically evaluated, and this revealed that the breast primary was consistent with the original biopsies. However, the right axillary lymph node revealed carcinoma with both neuroendocrine and squamous differentiation, ER 1%–2%, PR 1%–2%, and HER2 Neg (0 by IHC), and she was diagnosed with TNBC with neuroendocrine and squamous differentiation. Repeat PET imaging after surgery revealed ipsilateral supraclavicular lymphadenopathy and multiple new pulmonary nodules, and she was diagnosed with metastatic disease. Systemic therapy was aimed at addressing the more aggressive histology, TNBC. The tumor was found express the programmed death ligand 1, and treatment was initiated with nanoalbumin bound-paclitaxel (Abraxane, Abraxis BioScience, LLC, a Bristol-Myers Squibb Company, Los Angeles, USA) in combination with atezolizumab (Tecentriq, Genentech, a subsidiary of Roche, South San Francisco, USA) immunotherapy, based upon the FDA’s initial accelerated approval for atezolizumab as the first immunotherapeutic agent in this setting, based upon the phase III IMPASSION130 trial, which was later withdrawn.^2^

**Figure 1.**
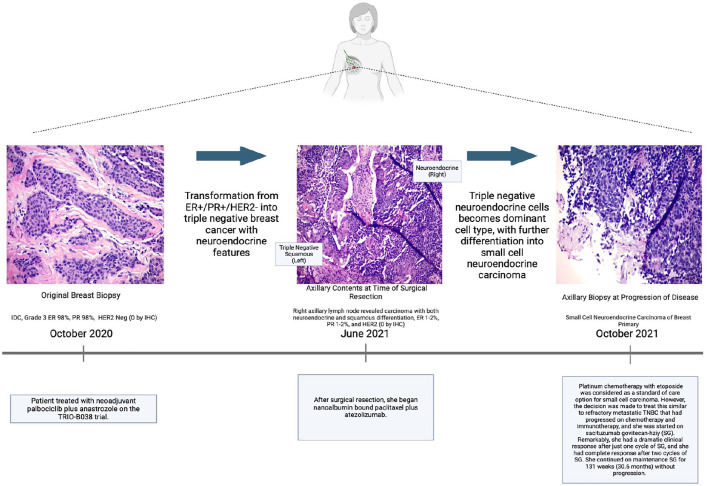
Patient timeline with treatment decisions and events correlated with changes in pathology. Ultimately, through a combination of pathology analysis and treatment-related responses, identifying neuroendocrine pathology of axillary disease. Source: (Created in BioRender (Fitzsimmons K. (2025) https://BioRender.com).

Unfortunately, after 4 months, the patient had a second rapid progression while on systemic therapy. Given the short time to progression and the neuroendocrine features seen at surgery, a repeat biopsy of the right axillary lymph node was performed. This demonstrated the transformation of her TNBC into small cell carcinoma (Figure 1). Platinum chemotherapy with etoposide was considered a standard-of-care option for small cell carcinoma. However, the decision was made to treat this similar to refractory metastatic TNBC that had progressed on chemotherapy and immunotherapy, and she was started on SG. Remarkably, she had a dramatic clinical response after just one cycle of SG, and she had a complete response after two cycles of SG. Her dose was reduced from 10 to 7.5 mg/kg for tolerability (given her fatigue, nausea/vomiting, and mild neutropenia). She remained on therapy until the progression of her disease in her liver two and a half years later with no evidence of disease for more than one and a half years after initiating SG. Given her long duration of therapy and side effects, the patient requested a “chemo holiday.” The patient experienced increased axillary swelling 8 weeks after this treatment pause. Remarkably, the subsequent re-initiation of SG led to rapid tumor regression. She eventually developed progression approximately two and a half years (30.6 months, 131.14 weeks) after initiating SG, whereas she had minimal durable response on prior systemic therapies.

Subsequently, she was treated with carboplatin and etoposide for four cycles, but she progressed in the axillary lymph nodes and liver. She then underwent two cycles of liposomal doxorubicin but experienced progression in the chest wall. She had palliative intent radiation therapy to the chest. She soon after opted for palliative care, hospice, and the End of Life Option Act for an aid-in-dying drug.

## Discussion

A particularly rare subtype of breast cancer is primary neuroendocrine neoplasms of the breast (PBNENs), which account for less than 0.1% of all BCs.^3^ While neuroendocrine differentiation may be observed in up to 20% of breast carcinomas, immunobiological markers and stains for neuroendocrine tumors (NETs) are not routinely used during BC workups due to their rarity.^4^ As such, much of what is known about these cancers comes from limited retrospective analysis and case series. Given the rarity of PBNENs, metastasis from another primary NET should be considered in the differential diagnosis.

The pathophysiology of a PBNEN is believed to occur during the carcinogenesis process, where neoplastic cells with neuroendocrine features arise from concurrent neoplastic epithelial progenitor cells, rather than from a purely neuroendocrine stem cell. This mixed endocrine and exocrine neoplasm hypothesis is further supported by the fact that many cases of PBNEN resemble the histology of known-type BCs, having co-expression of neuroendocrine cells and, most commonly, either luminal A or luminal B HER2-negative ductal carcinoma.^5^ In addition, unlike other neuroendocrine sites prone to benign hyperplastic transformation, such as pancreatic or lung NETs, there is very little evidence of neuroendocrine cells in benign breast masses, suggesting the PBNENs originate in a concurrent carcinogenesis process.^6^ Thus, favoring divergent differentiation (exocrine and endocrine) of a neoplastic epithelial progenitor cell versus a preexisting neuroendocrine stem cell.^5^

**Figure 2.**
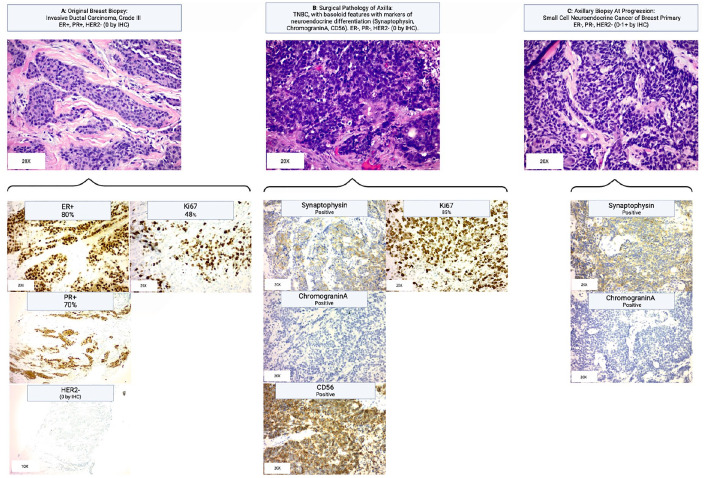
Histology of the series of biopsies which display the transformation of the original grade III IDC to small cell neuroendocrine carcinoma of breast primary, with additional stains further providing evidence of the neuroendocrine cell line’s development. (a) Original breast biopsy showing grade III IDC with stains indicating ER positive, PR positive, and Ki67. (b) Surgical pathology of axillary contents demonstrated triple-negative breast cancer: ER negative, PR negative, HER2 Neg (0 by IHC; stains not shown), and the emergence of a new basaloid cell line, differing from the original IDC squamoid line. Further staining shows positive synaptophysin and chromogranin A staining, both indicative of neuroendocrine cell types with further testing with CD56 indicative of the small cell neuroendocrine phenotype. (c) Additional axillary biopsy during the treatment course showing the small cell neuroendocrine cell as the dominant cell type, with synaptophysin and chromogranin A staining continuing to remain positive. Source: Created in BioRender (Fitzsimmons K. (2025) https://BioRender.com/w65u497). ER, estrogen receptor; HER2 Neg, human epidermal growth factor receptor 2 negative; IDC, invasive ductal carcinoma; PR, progesterone receptor.

This patient had the subtype of PBNEN known as primary SCNCB. Compared to other PBNEN subtypes, the small cell subtype has a more aggressive behavior and is associated with poorer outcomes. This closely resembles the well-studied behavior of commonly encountered small cell lung carcinomas which are rapidly proliferating and have an initially favorable response, but nearly universally relapse.^7^ Unlike the other PBNENs which tend to express ER and PR, the SCNCB subtype tends to have low hormone receptor expression and can fall into the larger category of TNBC.^8^ Thus, the “triple negative” nature of SCNCB and its aggressive nature lends its treatment to be similar to that of TNBC. In this case, the delayed presentation to medical providers for approximately 3 years may have contributed to significant tumor heterogeneity. This patient’s rapid clinical progression led to clinical suspicion that she did not have the expected response to therapy, which in combination with opportune biopsy samples, were key factors in identifying the several histologic transformations that took place; from ER+/PR+ disease to TNBC with neuroendocrine and squamous cell features, and ultimately to a small cell carcinoma of the breast (Figure 2).

On a molecular level, there are close matches between both SCLC and SCNCB.^9^ Of note, NETs of both lung and breast tend to have varying levels of overexpression of TROP2, a transmembrane glycoprotein that signals cell self-renewal, survival, and proliferation. TROP2 remains an interesting area of research for NETs. Interestingly, in neuroendocrine subtypes of lung cancer, high TROP expression is associated with lower patient mortality when compared to non-endocrine subtypes of lung cancer, such as adenocarcinoma or squamous, even if these subtypes are also expressing high levels of TROP2.^10^ This suggests a differential role for TROP2 in neuroendocrine subtypes. The role of anti-TROP2 agents for SCNCB is an area that still requires further research, with prior literature limited to tumor genomic profiling studies. Vranic et al.^11^ found high TROP2 protein expression in 21% of surgical tumor samples of neuroendocrine BC. Another tumor genomics study found 40% of breast NETs overexpressed topoisomerase-1, which is closely linked to TROP2 given that treatments that target TROP2 utilize a topoisomerase-1 inhibitor as the payload in the antibody-drug conjugate.^9^ These results suggest that some breast neuroendocrine cancers may have an amenable treatment response to TROP2 and topoisomerase-targeted treatments, such as SG.

**Figure 3.**
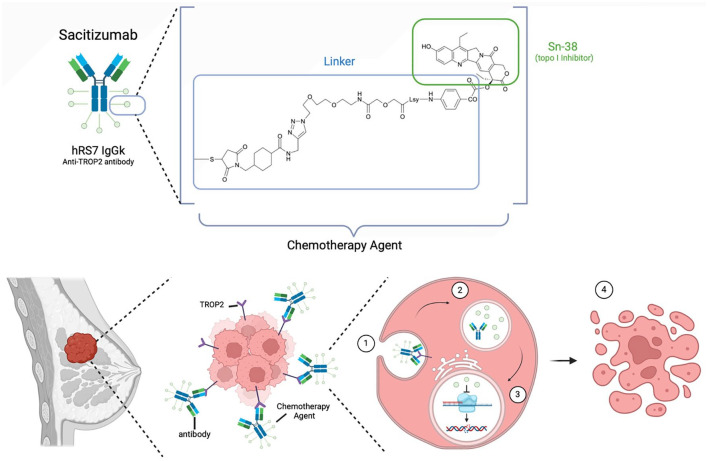
The structure and chemical profile of SG (Trodelvy). SG is an antibody-drug conjugate made up of an anti-TROP2 antibody known as hRS7 which has been linked to the chemotherapy agent Sn-38, a topoisomerase I inhibitor. The mode of action of SG includes antibody targeting of TROP2 expression on cancer cells, which are then (1) endocytosed into the tumor cell where (2) Sn-38 disassociates from its antibody conjugate before (3) entering the nucleus and halting transcription leading to (4) apoptosis of tumor cell. Source: Created in BioRender (Fitzsimmons K. (2025) https://BioRender.com/k47f388). SG, sacituzumab govitecan-hziy.

While there is limited data and literature on the treatment of SCNBC, the current standard treatment likely mimics treatment used for small cell carcinoma of other organs, such as SCLC. Typically, this is platinum chemotherapy combined with etoposide and immune checkpoint inhibition. Our patient already received taxane chemotherapy with immunotherapy prior to SG. SG was chosen because our patient had already received chemotherapy in combination with immunotherapy, and SG was approved for patients with metastatic TNBC. SG is an antibody-drug conjugate targeting the TROP2 protein in conjunction with a Sn-38 topoisomerase I inhibitor chemotherapy “payload” (Figure 3). The randomized phase III ASCENT trial found that patients with relapsed or refractory TNBC on SG had a nearly double medial overall survival versus those on physician’s choice single-agent chemotherapy (12.1 vs 6.7 months, respectively, hazard ratio 0.48, *p* < 0.001).^12^ Interestingly, we present a case of a patient with 30.6 months of response with SG compared to TNBC patients on ASCENT. Biomarker analysis from this trial revealed that the greatest benefit was seen in those who had medium or high Trop-2 expression, suggesting that TROP2 expression may correlate with response. SG as a second-line treatment in patients with extensive-stage SCLC has shown promise according to results from the ongoing basket trial, the multicohort phase II TROPiCS-03 trial (NCT03964727) presented at the 2023 ESMO Congress. At a median follow-up of 3.5 months (range, 0.6–9.4), evaluable patients treated in the ES-SCLC cohort (*n* = 26) achieved an overall response rate (ORR) of 29% (95% confidence interval (CI), 8%–58%).^13^ This response rate should be considered in a disease with low ORRs in the second-line setting and beyond. Response rates may be far different in small cell breast cancer (SCBC), given the difference in pathogenesis, since SCLC almost always arises due to repeated smoking insults to the lung tissue, but this needs to be addressed in clinical trials.^14^

## Conclusion

We believe this to be the first case where SG was used to treat a patient with SCNCB, and she had an essentially immediate and complete response with therapy for close to 2.5 years. Our encouraging clinical experience suggests that SG should be further studied for this indication. Further research is needed to determine whether TROP2 expression may play a role in the transformation of NETs of breast primary, and if this represents a potential therapeutic strategy with targeted TROP2 therapies. In conjunction, the high expression of topoisomerase-1 in breast NETs suggests that SG, a TROP2 inhibitor, and similar drugs in development may be ideal drugs to study for patients with SCNB.

## Declarations

**Ethics approval and consent to participate:** Not applicable.

**Consent for publication:** Written informed consent for publication of this case report, including any accompanying data or images, was provided by the patient.

**Availability of data and materials:** Not applicable.
